# Correction

**DOI:** 10.1080/20008066.2024.2355052

**Published:** 2024-06-14

**Fully remote intensive trauma-focused treatment for PTSD and Complex PTSD**

H. Bongaerts, Voorendonk, E. M., Van Minnen, A., Rozendaal, L., Telkamp, B. S. D., & de Jongh, A. (2022).

*Journal of Psychotraumatology, 13*(2), 2103287

https://doi.org/10.1080/20008066.2022.2103287

During preparation for another study, coding errors were discovered. These errors involved mistakenly coding some female participants as male. Further investigation showed that this coding error also affected some of the data used in this study. Several women were coded as male, which changed the outcomes of the study somewhat for the analyses in which gender was examined. Therefore, we have corrected the contents of the article. All corrections involve (only) the Results section:

1. The mean age of this group of participants was 37.16 years (SD = 11.82, age range 19–63), and 37 (50.7%) were female.

Now reads:

The mean age of this group of participants was 37.16 years (SD = 11.82, age range 19–63), and 62 (85%) were female.

2. Table 1 has been revised.

Now reads:

**Table 1.** Descriptives of the total sample of patients at baseline: Mean scores of the Clinician Administered PTSD Scale for DSM-5 (CAPS-5), PTSD Checklist for DSM-5 (PCL-5), and International Trauma Questionnaire (ITQ), age and sex, PTSD and Complex PTSD diagnosis, traumatic events according to the Life Events Checklist for DSM-5 (LEC-5), comorbidity, suicidal ideation.

	Total Sample				
Variable	*n*	M	SD	*Min*.	*Max*.
CAPS-5 pre-treatment	73	38.71	7.03	24	56
CAPS-5 post-treatment	73	13.12	11.70	0	44
CAPS-5 follow-up 0	57	15.63	14.70	0	55
PCL-5 pre-treatment	73	45.51	12.09	16	70
PCL-5 post-treatment	61	19.02	16.52	0	70
PCL-5 follow-up	62	21.81	18.40	0	67
ITQ-DSO pre-treatment	70	13.07	5.34	3	22
ITQ-DSO post-treatment	60	6.45	6.08	0	20
ITQ-DSO follow-up	63	7.29	6.60	0	21
Age	73	37.16	11.82	19	63
**Sex^1^**	***n***	**%**			
Male	11	15			
**PTSD at pre-treatment in accordance with CAPS-5^1^**	***n***	**%**			
Patients with PTSD	73	100			
**CPTSD at pre-treatment in accordance with ITQ^2^**	***n***	**%**			
Patients with Complex PTSD	33	47,1			
**LEC-5^1^**	***n***	**%**			
Physical abuse	64	87.7			
Sexual abuse	48	65.8			
Other traumatic events	44	60.3			
Life threatening situations such as accidents	37	50.7			
Natural disasters	6	8.2			
Warzone situations	5	6.8			
**One or more Comorbidity^1^**	***n***	**%**			
Depressive disorders	39	53.4			
Anxiety disorders	40	54.8			
Suicidal ideation^1^	n	%			
Reported moderate to high suicidal risk	19	26.0			

Note.

^1^ *n* = 73.

^2^ *n* = 70.

3.2. CAPS-5

The addition of predictors and covariates to the random intercept model improved the fit significantly, (Δχ²[6] = 189.25, p < .001). Adding a random slope for ‘measurement time point’ improved the fit of the model again (Δχ²[5] = 53.57, *p* < .001), which led to our main models 1 and 2 (see Table 3). The intercept for Model 1 (b0 = 35.62, SE = 1.51) represents the estimated mean value for the reference group at pre-treatment (reference group is ‘woman of average age, without Complex PTSD at pre-treatment and no missing data for the three instruments).

Now reads:

The addition of predictors and covariates to the random intercept model improved the fit significantly, (Δχ²[6] = 189.59, *p* < .001). Adding a random slope for ‘measurement time point’ improved the fit of the model again (Δχ²[5] = 53.37, *p* < .001), which led to our main models 1 and 2 (see Table 3). The intercept for Model 1 (b0 = 35.79, SE = 1.41) represents the estimated mean value for the reference group at pre-treatment (reference group is ‘woman of average age, without Complex PTSD at pre-treatment and no missing data for the three instruments).

Table 2 has been revised:

**Table 2.** Multilevel regression analyses of the Clinician Administered PTSD Scale for DSM-V (CAPS-5) by measurement moment, age and sex, and Complex PTSD.

Dependent variable: CAPS-5						
	Model 1			Model 2		
Predictor	b	SE	*p*	b	SE	*p*
Level 1						
Pre-treatment (intercept)	35.78	1.41	.00	35.58	1.44	.00
Post-treatment	-25.81	1.43	.00	-24.84	1.97	.00
Six months follow up	-23.15	1.76	.00	-22.62	2.45	.00
Level 2						
Male	1.27	2.27	.58	1.28	2.28	.58
Age	0.06	0.07	.38	0.06	0.07	.38
Complex PTSD	4.99	1.69	.004	5.43	1.79	.004
Participants with missing data	0.71	1.68	.68	0.71	1.69	.68
Interaction effect						
Post * Complex PTSD				-2.07	2.86	.47
Follow-up * Complex PTSD				-1.16	3.54	.74
	Variances (random effects)					
Pre-treatment (intercept)	30.69			30.67		
Post treatment	112.75			111.73		
Six months follow up	162.16			161.20		
Residual	12.39			12.36		
Level I: number of observations	195			195		
Level 2: number of participants	70			70		
	Compared to random intercept, random slopes model			Compared to Model 1		
Log likelihood ratio test						
Delta X^2^ (df)	53.37 (5)			0.62 (2)		
*p*	.001			.74		
ICC (random intercept only model)	0.00					

Note 1: Reference group (Intercept); Female, average Age, Completed all measurements, No Complex PTSD, at Pre-Treatment.

Note 2: Random Intercept and Random Slope For Measurement Moment for Models 1 and 2.

Table 3 has been revised:

**Table 3.** Multilevel regression analyses of the PTSD Checklist for DSM-5 (PCL-5) by measurement moment, age and sex, and Complex PTSD.

Dependent variable: PCL-5						
	Model 1			Model 2		
Predictor	b	SE	*p*	b	SE	*p*
Level 1						
Pre-treatment (intercept)	38.19	2.83	.00	37.01	2.17	.00
Post-treatment	-26.19	1.92	.00	-22.65	2.76	.00
Six months follow up	-23.02	1.92	.00	-21.62	2.79	.00
Level 2						
Male	3.86	4.40	.38	2.92	3.54	.41
Age	0.16	0.13	.24	-0.006	0.11	.096
Complex PTSD	11.03	3.21	.001	13.01	2.68	.00
Participants with missing data	2.89	3.25	.038	3.99	2.61	.13
Interaction effect						
Post * Complex PTSD				-7.51	4.02	.06
Follow-up * Complex PTSD				-3.14	4.15	.45
	Variances (random effects)					
Pre-treatment (intercept)	73.48			73.65		
Post treatment	218.88			201.27		
Six months follow up	216.75			214.89		
Residual	21.51			21.05		
Level I: number of observations	190			190		
Level 2: number of participants	70			70		
	Compared to random intercept, random slopes model			Compared to Model 1		
Log likelihood ratio test						
Delta X^2^ (df)	37.49			4.09		
*p*	<.001			.13		
ICC (random intercept only model)	0.131					

Note 1: Reference group (Intercept); Female, average Age, Completed all measurements, No Complex PTSD, at Pre-Treatment.

Note 2: Random Intercept and Random Slope For Measurement Moment for Models 1 and 2.

